# Associations and gender differences between OHI-seeking behaviors and eHealth literacy among Chinese university students

**DOI:** 10.1590/1980-220X-REEUSP-2023-0340en

**Published:** 2024-03-25

**Authors:** Jie Chen, Hua Tian

**Affiliations:** 1 Xinyang Normal University, School of Marxism, Xinyang, China.; 2 Xinyang Normal University, College of Life Science, Xinyang, China.

**Keywords:** Health Literacy, Health Information Management, Gender Identity, Students, Health Education, Letramento em Saúde, Gestão da Informação em Saúde, Identidade de Gênero, Estudantes, Educação em Saúde, Alfabetización en Salud, Gestión de la Información en Salud, Identidad de Género, Estudiantes, Educación en Salud

## Abstract

**Objective:**

to explore associations and gender differences between OHI-seeking (online health information seeking) behaviors and eHealth (electronic health) literacy among Chinese university students.

**Methods:**

Online questionnaires of eHealth literacy scale and OHI-seeking behaviors created in software Wenjunxing were used in this survey. Chi-squared tests, t-test, and Pearson correlation analysis were performed using SPSS for data analysis.

**Results:**

Among 5,383 participants, 72.4% were girls, 77.5% lived in rural areas, 51.2% majored in liberal arts, 76.6% with low education parents. The average C-eHEALS scores of boys and girls were 26.53 ± 5.861 and 26.84 ± 5.816, respectively, with no significant difference (P = 0.084). The top three OHI-seeking behaviors for boys and girls, as well as for the C-eHEALS low and high groups, were “finding information about physical exercises” “reading or sharing health information via social media” “finding information about nutrition and diet”, all of which had significant gender and eHealth literacy differences.

**Conclusions:**

Gender and eHealth literacy differences should be focused for intervention when developing and implementing eHealth intervention training for parents and adolescents in schools and communities.

## RESUMO

**Objetivo:**

Visa explorar associações e diferenças de gênero entre comportamentos de busca de OHI (busca de informações de saúde on-line) e letramento em e-Saúde (saúde eletrônica) entre estudantes universitários chineses.

**Métodos:**

Questionários on-line da escala de letramento em e-Saúde e comportamentos de busca de OHI criados no software Wenjunxing foram utilizados nesta pesquisa. Testes qui-quadrado, teste t e análise de correlação de Pearson foram realizados utilizando SPSS para análise de dados.

**Resultados:**

Dos 5.383 participantes, 72,4% eram meninas, 77,5% viviam em áreas rurais, 51,2% eram formados em artes liberais, 76,6% tinham pais com baixa escolaridade. As pontuações médias do C-eHEALS de meninos e meninas foram 26,53 ± 5,861 e 26,84 ± 5,816, respectivamente, sem diferença significativa (P = 0,084). Os três principais comportamentos de busca de IHO para meninos e meninas, bem como para os grupos baixo e alto do C-eHEALS, foram “encontrar informações sobre exercícios físicos”; “ler ou compartilhar informações de saúde através das redes sociais”; “encontrar informações sobre nutrição e dieta”, todos com diferenças significativas de gênero e de letramento em e-Saúde.

**Conclusões:**

As diferenças de gênero e de letramento em e-Saúde devem ser focadas na intervenção ao desenvolver e implementar formação de intervenção em e-Saúde para pais e adolescentes em escolas e comunidades.

## RESUMEN

**Objetivo:**

Explorar las asociaciones y las diferencias de género entre el comportamiento de búsqueda de OHI (información sanitaria en línea) y los conocimientos sobre e-Salud (salud electrónica) entre estudiantes universitarios chinos.

**Métodos:**

Se utilizaron cuestionarios en línea de la escala de conocimientos en e-Salud y conductas de búsqueda de OHI creadas con el software Wenjunxing. En el análisis de los datos, se utilizó el programa SPSS para llevar a cabo las pruebas de chi-cuadrado, pruebas t y análisis de correlación de Pearson.

**Resultados:**

De los 5.383 participantes, el 72,4% estaba formado por muchachas, el 77,5% vivía en zonas rurales, el 51,2% era licenciado en Humanidades y el 76,6% tenía padres con bajo nivel educativo. Las puntuaciones medias del C-eHEALS para chicos y chicas fueron 26,53 ± 5,861 y 26,84 ± 5,816, respectivamente, sin diferencias significativas (P = 0,084). Las tres conductas principales de búsqueda de la OHI para muchachos y muchachas, así como para los grupos de C-eHEALS bajo y alto, fueron “encontrar información sobre ejercicio físico”; “leer o compartir información sobre salud a través de las redes sociales”; “encontrar información sobre nutrición y dietas”, todos ellos con diferencias significativas de género y de aprendizaje sobre e-Salud.

**Conclusiones:**

Las diferencias de género y de conocimientos en e-Salud deben centrarse en la intervención a la hora de desarrollar e implementar la formación en cibersalud para padres y adolescentes en escuelas y comunidades.

## INTRODUCTION

Health information seekers are increasingly using online health information to find answers to health related questions^(1)^. The ability to find, evaluate, and use online health information may be affected by an individual’s electronic health literacy (eHealth literacy) level^(2)^, which ensures the effective use of health information. eHealth literacy is defined as “the ability to seek, discover, understand, and evaluate health information from electronic sources, and apply the acquired knowledge to solving or solving health problems.”^(3)^ Studies showed that people with high eHealth literacy were more likely to seek health information and health knowledge^(4)^, participate in health behaviors and screening practices^(5)^, and had better self-management skills and interactions with doctors^(6)^.

During the COVID-19 pandemic, eHealth literacy may be an important factor in raising awareness of pandemic infectious diseases and forming healthy living habits among Korean middle school students^(7)^. Chinese college students had low eHealth literacy, and women were lower than men^(8)^. Under the condition of similar educational levels, Taiwan female college students had lower functional eHealth literacy than men^(9)^. There were also studies that reported women were associated with limited functional health literacy^(10)^, which may be due to their low educational level^(11)^. However, some studies had reported that there was no significant relationship between gender and eHealth literacy^(12)^, which was related to the increase in online health information seeking among adolescents^(13)^.

Thus, the following hypothesis is proposed:

H1: There are significant gender differences in eHealth literacy among Chinese college students, which is helpful when building future strategies for enhancing eHealth literacy.

A recent study showed that compared to junior high school girls, junior high school boys used Internet more often to seek health information. The health content searched by junior high school girls included “weight loss” and “beauty and health”. The health content searched by high school girls included “mental health”, “weight loss”, and “beauty and health”. The health content searched by high school boys included “sexual knowledge”. It can be seen that girls were more interested in weight loss, beauty, and health than boys, while high school students searched multiple categories of online health information more frequently than junior high school students^(13)^.

With the development of the Internet and the application of 5G smart phone, there are more and more online health information and electronic health services. However, most adolescents still have difficulties in finding and distinguishing reliable online health information sources^(12)^. Studies had shown that many adolescents had low eHealth literacy, and their ability to search and evaluate health information online was negatively affected by low eHealth literacy^(14)^. Adolescents who were able to use reliable online health information sources tend to have higher eHealth literacy^(15)^.

Thus, the following hypothesis is proposed:

H2: There are significant gender differences in OHI-seeking behaviors among college students in China, which may provide reference for improving OHI-seeking capabilities.
H3: There are significant associations between C-eHEALS and OHI-seeking behaviors, which can contribute to future intervention trainings.

Therefore, this study investigated the associations and gender differences between OHI-seeking behavior and eHealth literacy among Chinese college students, so as to explore diversified intervention measures. The findings can help parents and adolescents in schools and communities to develop and implement eHealth intervention training.

## METHOD

### Design of Study

A non-experimental comparative study used quantitative research methods. The study used online questionnaires of eHealth literacy scale and OHI-seeking behaviors, which were created in Wenjunxing (https://www.wjx.cn/) software. The participant recruitment happened following these steps: the researchers sent the questionnaire link to a group of WeChat counselor teacher users from Xinyang Normal University and asked them to share it with students to recruit voluntary participants. The questionnaire was anonymous. Voluntary participant college students can freely and independently fill out questionnaires on their computers or smartphones. Finally, a total of 5,672 college students were recruited.

### Sample Definition

University students were recruited from Xinyang Normal University in China, with the stated goal of examining associations and gender difference between eHealth literacy and OHI-seeking behaviors among Chinese university students. The investigation was conducted from October to December 2022.

### Selecton Criteria

All participants were native Mandarin speakers aged 18–23 years old, from freshmen to seniors. Their majors are science, liberal arts, engineering and arts. Participants who did not complete the questionnaire were excluded (n = 289), and finally 5,383 (response rate: 94.90%) university students were selected as respondents.

### Data Collection Instrument

Data were collected using online questionnaires in Wenjuanxing software. eHealth literacy scale was used the Chinese version (C-eHEALS). According to C-eHEALS average score of 26 (n = 5,383), boys and girls were divided two groups of C-eHEALS-high (≥26, n = 2,553) and C-eHEALS-low (<26, n = 2,830). About OHI-seeking behaviors, boy and girl university students were asked to answer if they had any of the following behaviors while surfing the Internet in the past 12 months. According to their responses the frequency of eleven OHI-seeking behaviors was counted.

### Data Analysis

Reliability and validity of two questionnaires were verified through Kaiser–Meyer–Olkin (KMO) test and Cronbach’s Alpha. The two questionnaires have been verified and have high reliability and validity^(16)^. Chi-squared tests, *t*-test, Pearson correlation analysis were used to compare the differences between boys and girls, as well as descriptive statistics, such as frequency, percentage, average, standard deviation. SPSS v20 (IBM, Armonk, NY, United States) was used for data analysis.

### Ethical Aspects

This study was performed in compliance with the Helsinki Declaration guidelines. All procedures relevant to study participants were approved by the Ethics Committee of Xinyang Normal University (XFEC-2023–025). Participation was voluntary; participants were informed of the study objective and context and provided their written informed consent regarding privacy and information management policies.

## RESULTS

### Demographic Characteristics

Among the 5,383 university students, 72.4% were girls, and 27.6% were boys. 77.5% lived in rural areas and 22.5% lived in town. Half (51.2%) of the students majored in liberal arts and 28.0% majored in science. 76.6% university students’ parental education level was middle school education or below. Only 0.3% of them had a PhD or a master’s degree. Thus, participant university students had some notable characteristics: mainly girls, from rural areas, who were majoring in liberal arts, with parents who had low education level (Table 1).

**Table 1:** Participant characteristics (n = 5,383) – Xinyang, Henan, China, 2023.

Characteristics		Frequency	Proportion (%)
Gender			
	Boys	1,488	27.6
	Girls	3,895	72.4
Residence			
	Town	1,212	22.5
	Rural	4,171	77.5
Major			
	Liberal arts	2,756	51.2
	Science	1,509	28.0
	Engineering	558	10.4
	Art	560	10.4
Parental education level			
	PhD/Master	14	0.3
	University/College	1,245	23.1
	Middle school	4,124	76.6

### Gender Difference in C-eheals

From Table 2, the Kaiser-Meyer-Olkin (KMO) and Cronbach’s alpha of C-eHEALS were 0.916 and 0.934, respectively, showing excellent reliability. Significant differences between boy and girl C-eHEALS were certified for eight C-eHEALS items (*P* < 0.001). The average boy and girl C-eHEALS scores were 26.53 ± 5.861 and 26.84 ± 5.816, respectively. There was no significant difference between the two groups (*P* = 0.084). However, it should be noted that there are significant gender differences among the eight items of eHealth literacy.

**Table 2:** Gender analysis of C-eHEALS (n = 5,383) – Xinyang, Henan, China, 2023.

Items	Characteristics	Boys	Girls	*P* value
C-eHEALS1	I know what health resources are available on the Internet	3.46 ± 0.916	3.21 ± 0.790	<0.001
C-eHEALS2	I know where to find helpful health resources on the Internet	3.42 ± 0.948	3.14 ± 0.824	<0.001
C-eHEALS3	I know how to find helpful health resources on the Internet	3.45 ± 0.951	3.18 ± 0.828	<0.001
C-eHEALS4	I know how to use the Internet to answer my questions about health	3.48 ± 0.944	3.24 ± 0.849	<0.001
C-eHEALS5	I know how to use the health information I find on the Internet to help me	3.57 ± 0.922	3.36 ± 0.830	<0.001
C-eHEALS6	I have the skills I need to evaluate the health resources I find on the Internet	3.56 ± 0.962	3.37 ± 0.911	<0.001
C-eHEALS7	I can tell high quality health resources from low quality health resources online	3.64 ± 0.945	3.49 ± 0.893	<0.001
C-eHEALS8	I feel confident about using information from the Internet to make health decisions	3.53 ± 0.956	3.24 ± 0.902	<0.001
Total		26.53 ± 5.861	26.84 ± 5.816	0.084
Kaiser-Meyer-Olkin (KMO)		0.916		
Cronbach’s alpha		0.934		

Note. P-values yielded by t-test.

Thus, the result does no support H1.

### Gender Difference in Ohi-seeking Behaviors

OHI-seeking behaviors of boys and girls in the past 12 months were showed in Table 3. Among the eleven OHI-seeking behaviors commonly used by college students, except “finding health or medical information (*P* = 0.148)” “writing and sharing health information via social media (*P* = 0.056)” and “purchasing health care products online (*P* = 0.280)”, the other eight OHI-seeking behaviors had significant gender differences between girls and boys (*P* < 0.001). Comparing OHI-seeking behaviors of boys and girls based on their responses, boys’ favorite OHI-seeking behavior was searching for physical exercises online, while girls’ was searching for nutrition and diet online. However, the top three OHI-seeking behaviors among boys and girls were “finding information about physical exercises” “reading or sharing health information via social media” “finding information about nutrition and diet”.

**Table 3:** Gender differences in OHI-seeking behaviors (n = 5,383) – Xinyang, Henan, China, 2023.

OHI-seeking behaviors (multiple choice)	Boys (n = 1,488)		Girls (n = 3,895)		*P* value
Finding information about hospitals or doctors	426	28.63	936	24.03	<0.001
Finding information about physical exercises	769	51.68	1,487	38.18	<0.001
Finding information about how to stop smoking	137	9.21	94	2.41	<0.001
Finding health or medical information	383	25.74	1,079	27.70	0.148
Finding information about how to stop drinking	81	5.44	59	1.51	<0.001
Reading or sharing health information via social media	585	39.31	1,878	48.22	<0.001
Finding information about nutrition and diet	561	37.70	1,916	49.19	<0.001
Writing and sharing health information via social media	171	11.49	379	9.73	0.056
Attending a specific disease internet community	38	2.55	47	1.21	<0.001
Purchasing health care products online	117	7.86	273	7.01	0.280
Online reservation of health care projects	51	3.43	71	1.82	<0.001

Note. P-values yielded by chi-squared test.

OHI-seeking behaviors refer to those you have experienced while surfing the internet in the past 12 months.

Thus, the result supports H2.

### Association Between C-eheals and Ohi-seeking Behaviors

As shown in Table 4, there were significant positive associations between C-eHEALS and OHI-seeking behaviors excluding two items, namely “finding information about nutrition and diet” and “purchasing health care products online”.

**Table 4:** Pearson correlation analysis (n = 5,383) – Xinyang, Henan, China, 2023.

Characteristics	C-eHEALS
Finding information about hospitals or doctors	0.083**
Finding information about physical exercises	0.073**
Finding information about how to stop smoking	0.091**
Finding health or medical information	0.037**
Finding information about how to stop drinking	0.060**
Reading or sharing health information via social media	0.040**
Finding information about nutrition and diet	0.001
Writing and sharing health information via social media	0.069**
Attending a specific disease internet community	0.046**
Purchasing health care products online	0.023
Online reservation of health care projects	0.033*

Note. **indicates a significant correlation at the 0.01 level (bilateral);

*indicates a significant correlation at the 0.05 level (bilateral).

Social media, for example Weibo and WeChat.

As shown in Table 5, the favorite three OHI-seeking behaviors were “finding information about physical exercises (*P* < 0.001)”, “reading or sharing health information via social media (*P* = 0.003)”, and “finding information about nutrition and diet (*P* = 0.021)” among both the C-eHEALS-low and C-eHEALS-high groups. Although the three OHI-seeking behaviors were favorite for the C-eHEALS-low group, the proportion of participants was significantly lower than that of the C-eHEALS-high group. There were significant associations between C-eHEALS and OHI-seeking behaviors except for the last four OHI-seeking behaviors in Table 5.

**Table 5:** Association between C-eHEALS and OHI-seeking behaviors (n = 5,383) – Xinyang, Henan, China, 2023.

OHI-seeking behaviors (multiple choice)	C-eHEALS-low (n = 2,830)		C-eHEALS-high (n = 2,553)		*P*
Finding information about hospitals or doctors	674	23.82	688	26.95	0.008
Finding information about physical exercises	1,101	38.90	1,155	45.24	<0.001
Finding information about how to stop smoking	97	3.43	134	5.25	0.001
Finding health or medical information	730	25.80	732	28.67	0.018
Reading or sharing health information via social media	1,241	43.85	1,222	47.87	0.003
Finding information about nutrition and diet	1,260	44.52	1,217	47.67	0.021
Writing and sharing health information via social media	253	8.94	297	11.63	0.001
Finding information about how to stop drinking	65	2.30	75	2.94	0.140
Attending a specific disease internet community	42	1.48	43	1.68	0.556
Purchasing health care products online	202	7.14	188	7.36	0.749
Online reservation of health care projects	64	2.26	58	2.27	0.980

Note. P-values yielded by chi-squared tests.

Social media, for example Weibo and WeChat.

Thus, the result supports H3.

## DISCUSSION

eHealth literacy was defined by Norman CD in 2006 and initially consisted of six components: traditional literacy, health literacy, information literacy, scientific literacy, media literacy, and computer literacy^(2)^. There are two commonly used eHealth literacy assessment scales, one is an 8-item scale^(17)^, and the other is a 12-item scale included three levels of functional, interactive, and critical eHealth literacy^(18)^. At the most basic level, functional eHealth literacy referred to basic reading and writing skills, as well as basic knowledge of health conditions and health systems. Interactive eHealth literacy referred to communication and social skills that were used to abstract information and derive meaning from different forms of communication. The highest level of critical eHealth literacy was based on functional and interactive literacy, involving the most advanced cognitive skills that were used to critically analyze information, identify the quality of health websites, and used high quality information to make informed decisions about health^(19)^. College students with higher critical eHealth literacy participated in health promotion activities better than those with functional and interactive eHealth literacy^(20)^.

eHealth literacy plays an important role in the daily lives of young people. eHealth literacy can promote individual health behaviors, such as physical exercises and balanced nutrition. College students with advanced eHealth literacy skills and computer skills can utilize more effective online search strategies and identify high-quality health information resources. College students with high levels of eHealth literacy prefer search engines, such as Baidu and TikTok applications, face-to-face video interviews, specific health websites, WeChat or QQ social software, and online encyclopedias. Although college students in the low level eHealth literacy group also preferred the above methods to obtain health information, the proportion of participants was significantly lower than that in the high level group^(21)^.

Taiwanese college students with high functional eHealth literacy were more likely to engage in less unhealthy food consumption behaviors, and women with high interactive eHealth literacy were more likely to have a balanced diet^(22)^. If a college student has a low level of electronic health literacy, he or she may make poor health decisions due to incorrect information or misinformation (deliberate dissemination of misinformation) when searching for health information online. A study comparing the health information acquisition behaviors of young and older adult college students found that young college students (18–22 years old) often used websites to solve health problems, while elderly college students (55–72 years old) used communication software to convey health information^(23)^. If a college student has a low level of eHealth literacy, he or she may make poor health decisions due to error information (incorrect information) or wrong information (intentional dissemination of incorrect information) when searching for health information online^(24)^.

For adults, women in Kuwait exhibit a higher average eHEALS than men^(25)^. Compared to people with lower eHealth literacy, middle-aged Korean people with higher eHealth literacy were mainly: older, female and with higher educational qualifications and higher levels of digital skills^(26)^. American adult women were more likely than men to seek health information online and had mobile applications related to health. The main driving factors for women to use Internet were interpersonal and educational use, while entertainment and leisure were the main driving forces for men^(27)^.

In terms of online health information search, researches focus on online health search for specific diseases, such as cancer, acute coronary syndromes, cyberchondria, and fertility barriers. The driving factors and comprehensive models of online health information quality, as well as the relationship between online health search and psychological health^(28)^, such as depression, anxiety, and well-being, involve children, adolescents, adults and older adults.

College students’ OHI-seeking mainly focuses on websites, social media, specific health information of obesity and chronic diseases, attitudes towards OHI-seeking and social support, and using obtained OHI-seeking to improve mental health and other health conditions^(29)^. Our study fills in the gender difference between college students’ OHI-seeking behaviors and eHealth literacy. College students with different genders had different levels of eHealth literacy, and gender had a significant impact on college students’ OHI-seeking behaviors. In the future, when developing and implementing eHealth intervention training for parents and adolescents in schools and communities, eHealth literacy should be used as a pre-test/post-test evaluation measure, and then training resources and measures with gender differences are separately designed to ensure the optimal interventions.

Limitations should not be ignored. First, as a cross-sectional design, this work cannot determine the study variables’ causality. Second, participants from a single university in China cannot provide an overview of OHI-seeking behaviors among Chinese university students, despite thousands of participants from different grades and majors. Third, there may be some biases in the results because of the self-reported questionnaire. Nevertheless, the heterogeneity of participating college students in our study can still provide insights into their OHI-seeking behaviors and eHealth literacy. Further research should focus on the intervention mechanisms of OHI-seeking and eHealth literacy based on gender differences, so as to provide appropriate and specific interventions to predict and improve health status.

## CONCLUSION

A total of 5,383 university students aged 18–23 years old participated in the survey. Most of them were girls (72.4%) and majored in liberal arts (51.2%) from rural areas (77.5%), and their parental educational level were middle school education or below (76.6%). The average C-eHEALS scores for boys and girls were 26.53 ± 5.861 and 26.84 ± 5.816, respectively, with no significant difference between the two (*P* = 0.084). The top three OHI-seeking behaviors for boys and girls, as well as for the C-eHEALS low and high groups, were “finding information about physical exercises” “reading or sharing health information via social media” “finding information about nutrition and diet”, all of which had significant gender and eHealth literacy level differences. Therefore, when developing and implementing eHealth and OHI-seeking intervention training for parents and adolescents in schools and communities, it is very important and necessary to provide gender differentiated training resources and methods to ensure the best intervention.
